# Are Biophilic-Designed Site Office Buildings Linked to Health Benefits and High Performing Occupants?

**DOI:** 10.3390/ijerph111212204

**Published:** 2014-11-26

**Authors:** Tonia Gray, Carol Birrell

**Affiliations:** 1 Centre for Educational Research, School of Education University of Western Sydney, Penrith 2751, Australia; 2 School of Education, University of Western Sydney, Penrith 2751, Australia; E-Mail: c.birrell@uws.edu.au

**Keywords:** biophilic design, site office, productivity, collaboration, well-being, stress

## Abstract

This paper discusses the first phase of a longitudinal study underway in Australia to ascertain the broad health benefits of specific types of biophilic design for workers in a building site office. A bespoke site design was formulated to include open plan workspace, natural lighting, ventilation, significant plants, prospect and views, recycled materials and use of non-synthetic materials. Initial data in the first three months was gathered from a series of demographic questions and from interviews and observations of site workers. Preliminary data indicates a strong positive effect from incorporating aspects of biophilic design to boost productivity, ameliorate stress, enhance well-being, foster a collaborative work environment and promote workplace satisfaction, thus contributing towards a high performance workspace. The longitudinal study spanning over two years will track human-plant interactions in a biophilic influenced space, whilst also assessing the concomitant cognitive, social, psychological and physical health benefits for workers.

## 1. Introduction

“Study Nature, love nature, stay close to nature. It will never fail you”.

*—Frank Lloyd Wright*

The positive effects of nature, especially plants, upon human health and well-being, has been extensively researched and documented [1,2,3,4,5,6,7,8,9,10,11,12]. Data outlining the benefits of human-nature contact include: stress reduction, healing, attention restoration, and the development of perceptual and expressive skills, as well as cognitive, imaginative and social capacity [13,14,15,16,17,18,19,20]. However, Elings [3] posits that little is known about the people-plant interactions or the mechanisms behind what some people refer to as horticultural therapy or nature based interventions [21,22]. More importantly, methodological limitations of previous studies reduce their usefulness as evidence-based research. Most research in this area has been strongly psychological, studying cognitive processes [23], the emotions [24,25], and well-being [26,27]. A recent extensive review [28] on the physiological effects of experiencing outdoor nature reported significant positive effects; however, most studies were short duration; took place outdoors, in forests, gardens or wildlife reserves; and were located in Japan, Europe or the USA.

According to Harvard biologist, E. O. Wilson [29,30], we are biologically drawn to nature. In essence, we are hard-wired to prefer natural settings, yet in our modern industrialised societies, we spend on average 90% of our time indoors in built environments, most often in cities [31,32]. These artificial settings seldom offer contact with nature and are not generally designed on natural principles. In contrast, biophilic design [33] incorporates such features as indoor-outdoor connections, natural ventilation and materials, extensive natural lighting, views of the outdoor landscapes, courtyards, natural landscaping, water features and interior designs that mimic shapes and forms found in nature. Research indicates that biophilic design enhances human well-being by fostering connections between people and nature in the modern built environment. The theory of biophilia provides a comprehensive framework for understanding human-nature relationships [34] and is articulated within nine unique “biophilic expressions” (see Table 1).

**Table 1:** A typology of values of nature [35].

Value	Definition
Aesthetic	Physical attraction and appeal of nature
Dominionistic	Mastery and control of nature
Humanistic	Emotional bonding with nature
Moralistic	Ethical and spiritual relation to nature
Naturalistic	Exploration and discovery of nature
Negativistic	Fear and aversion of nature
Scientific	Knowledge and understanding of nature
Symbolic	Nature as a source of language and imagination
Utilitarian	Nature as a source of material and physical benefit

Note: Adapted from Kellert, by Meltzer and colleagues [35].

## 2. Biophilic Design in Modern Architecture

Stephen Kellert [34] as a leading academic, has argued the degradation of natural systems is due to his dominant approach to architectural design. He believes the built environment has exacerbated human separation from the natural world. Biophilic design is an attempt to redress this imbalance and bring nature back into architecture by incorporating six key features: (1) environmental features; (2) natural shapes and forms; (3) natural patterns and processes; (4) light and space; (5) place-based relationships; and (6) evolved human-nature relationships [8,31]. Although a relatively new concept within contemporary architecture, biophilic design incorporates considerations of human health, ecology and sustainability principles. Proponents such as Almusaed and Asaad [36] advocate that buildings should be designed to incorporate the following design principles: (1) energy, activity and thermal comfort; (2) indoor/outdoor nature contact; (3) functional light and airy spaces; and (4) green building elements and energy saving components.

## 3. The Impact of Plants within the Built Environment

Nature writer Wendell Berry [37] coined the term “non-place” to refer to those settings lacking vitality and organic connectedness. Ostensibly, “dead” places constitute an ever-increasing proportion of our daily lives occupied within these sterile ‘non-places’. Recent research conducted by Burchett and colleagues [1] examined the effects of plant presence on negative mood states in building occupants. Their research was the first empirical study to use internationally validated psychological measures for assessing the potential benefits of indoor plants. The presence of plants correlates positively with worker productivity [38] as well as large reductions in negative mood states and levels of stress among building occupants [1,2,39,40,41,42]. Potted plants can improve indoor air quality [43] for building occupants, but of particular interest, Burchett, *et al.* revealed that just one plant within the workspace can significantly enhance staff morale and simultaneously promote well-being and improve performance [1].

Their seminal research focused on the benefits of potted plants in reducing air pollution indoors. Plants played a central role in ameliorating volatile organic compound (VOCs) emitted from plastic or synthetic materials (such as furnishings, furniture, and equipment like computers and photocopiers), and CO_2_ from occupants’ breathing. Cleaner air has also been found to have a causal relationship with better cardiovascular health and mental acuity [42,43].

Compelling evidence is mounting to encourage the incorporation of green spaces in work sites; this proposed two-year collaborative project between University of Western Sydney (UWS) and Brookfield Multiplex (BM), one of Australia’s largest construction companies, will examine if selected variables such as: a bespoke open plan site shed space; natural lighting and ventilation; introduction of plants; and collaborative work spaces have an impact on health and well-being.

### The Case Study Site

On each BM building site, the most essential component is the site office, adjoining the building site and serving as a temporary workplace for site managers who oversee the project. Although temporary, site managers move from one site office to another; thus the buildings constitute a long-term setting for their professional lives. In other words, “temporary” becomes “permanent” across different work sites.

BM is a partner of 202020 Vision [44] and supports the program because of their shared understanding that green spaces can have a significant and positive impact on building occupants. As an organization, BM’s goal is to create smarter, high performance buildings. In the words of Lauren Haas, Australasian Sustainability Manager at BM:

“Our goal with this project is to improve our site office environments which is an element of our broader business agenda of creating high performance site offices... because evidence tells us they make our people happier, healthier and more productive”.

In the first month of the study, the case study commenced with a “voluntary Saturday working bee” involving workers from a wide cross section of employees from apprentices through to upper management. This was a novel approach to work assignment, with the goal to empower workers and break down everyday hierarchy in the company to increase collaboration and solidarity.

**Figure 1:**
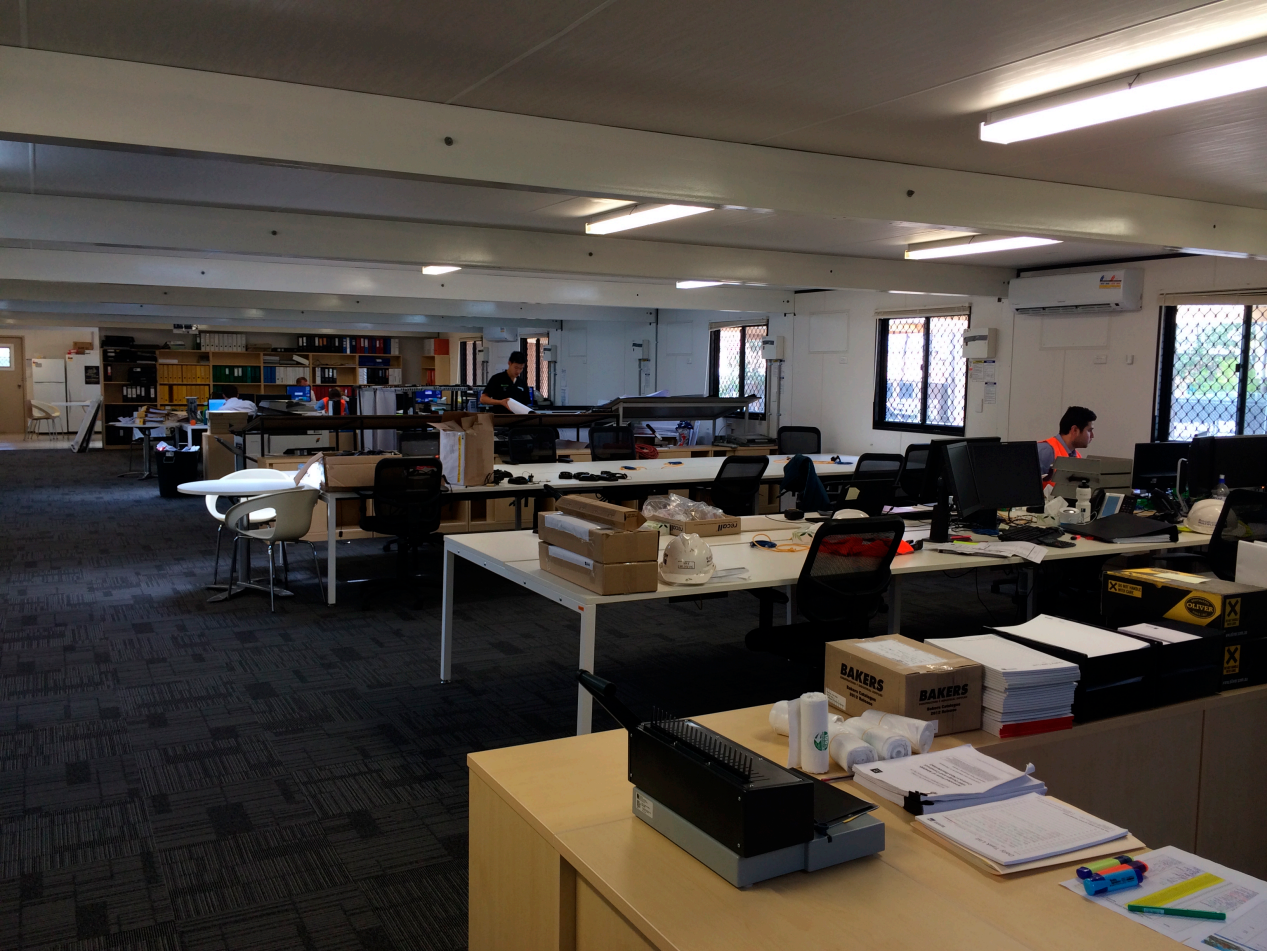
Bespoke open plan site office before plant arrival.

The bespoke workplace design of the biophilic site office (see Figure 1) was initiated by Lauren Haas.

**Figure A1:**
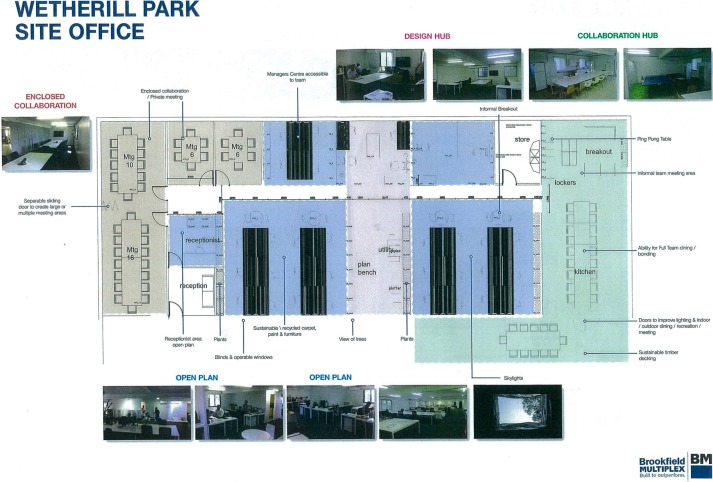
Bespoke site office at Brookfield Multiplex Wetherill Park.

The following characteristics defined the site office (see Appendix Figure A1):

- Four specific purpose spaces, including: (1) collaboration hub; (2) design hub; (3) enclosed collaboration area; and (4) open plan area.
- Skylights to allow for natural light.
- Recycled and sustainable carpet and furniture.
- Blinds and operable windows with views of trees.
- Recycled timber decking.
- Doors to improve lighting and ventilation.
- Extended kitchen with a breakout area for recreation and informal team meetings.
- 12 plant boxes at eye height between large rows of decks.
- 2 floor-to-ceiling plant walls.
- Herb tower for the verandah.

It can be seen that the design outlined here has incorporated certain biophilic design principles as articulated by both Kellert [8,31] and Almusaed and Asaad [36]. In particular, the considerably larger size of the site shed workplace and open space collaborative design aspects were seen to be deliberately addressing such biophilic design factors as “light and space” and “place-based relationships” emphasised by Kellert [8,31] as well as “energy, activity and thermal comfort” and “functional light and airy spaces” of Almusaed and Asaad [36].

## 4. Methodology

The aim of the two-year longitudinal study is to examine the impact of purposely introduced plants into a retrofitted site office shed which incorporates elements of biophilic design. Against this backdrop, our paper provides the initial findings from the first phase of a longitudinal two-year study (2014–2016). The site office workers are *in situ* for 24 months renovating a major suburban shopping mall in western Sydney, both quantitative and qualitative data will be obtained. The long-term research project incorporates a mixed-methods research design. Quantitative data for the longer-term project will be obtained from on-line survey results Connection to Nature Scale (CNS) [45], employee absentee records and self-reported health and well-being data. The emphasis in this paper is on the qualitative data obtained from interviews, observation, photographs and video footage. The data collection was initiated with the working bee and will continue at regular six-month intervals along the 24-month project. The primary research question underpinning this study is, what are the short-term wellbeing and perceptions of the working environment for workers in a retrofitted biophilic designed site office shed?

The preliminary data collection points during the first three months of this study are displayed in Table 2.

**Table 2:** Data collection up to May 2014.

Phase	Description
Phase 1	Preparatory meetings with key stakeholders prior to biophilic fit-out.
Phase 2	Qualitative data obtained from the working bee during brief interviews, observations, photographs, and video analysis of site workers.
Phase 3 (ongoing)	Qualitative data from interviews with randomly selected workers following biophilic refit of the site office.

### 4.1. Phase 1: Planning Stage

Several planning meetings were held with overall site manager and foreman of the site, 202020 Vision staff and Nursery and Garden Industry of Australia (NGIA) representative Matt Carroll, who were providing the plants for the retrofit. Discussions concerned the type of plants themselves as suitable for this environment (several varieties; little natural light required; must be hardy and not need constant watering; the density of plants throughout the office). The final plant selection included:

- Peace/Madonna Lily (Spathiphyllum species)
- Mother in Laws Tongue/ Snake Plant (Sansevieria species)
- Zanzibar Gem (Zamioculcas zamiifolia)
- Cast Iron Plant (Aspidistra)
- Grey Star (Ctenanthe setosa)

**Figure 2:**
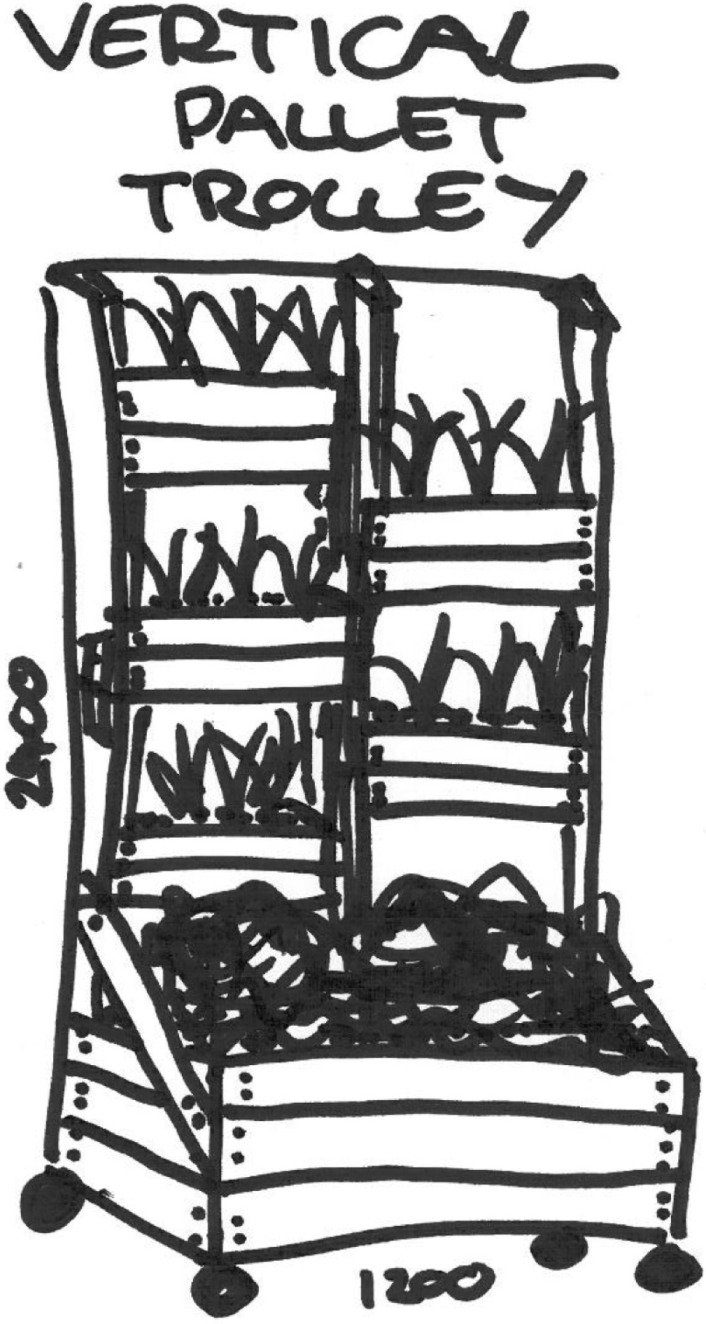
Concept design Vertical Pallet Trolley.

Decisions concerning what materials the plants would be seated in and exactly where in the site office they were to be located were important considerations. A biophilic approach features recycling natural materials wherever possible, so construction used wood pallets (see Figure 2), readily available on site and literally cost free.

Vertical pallets had to be easy to assemble on the designated working bee day with staff, friends and family invited to the site to participate in constructing the pallets, choosing and planting the plants and placing them in the designated spots in the site office. An underlying premise was that a model of easy-to-construct, cheap and attractive pallets could be adopted in a home context; thus, not only does the workplace or company model the use of recycled materials and greening of the workplace, but also encourages greening of the workers’ homes as a flow-on effect. This pattern of modeling and transferable techniques develops in a new direction the overall Sustainability Plan for BM, while incorporating all six elements of biophilic features [31].

### 4.2. Phase 2: Working Bee Overview

The study commenced with a Saturday working bee organised for site office workers (n = 17), their partners (n = 6) and children (n = 3). The primary intent of the exercise was to foster collaborative and group ownership of the project. Qualitative interviews, observation, photographs and video footage were obtained during the working bee proceedings.

**Figure 3:**
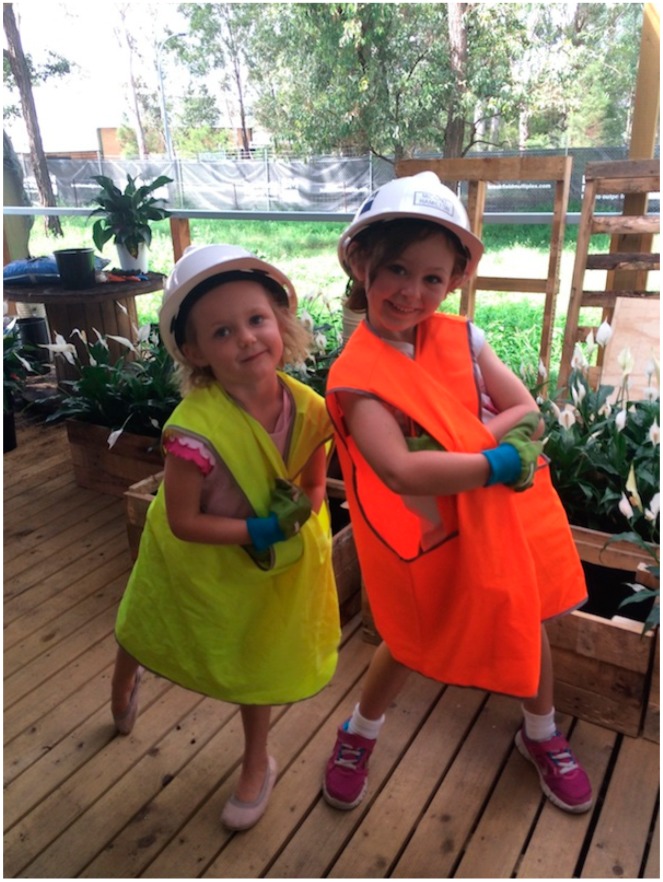
Children actively participating with their parents in the working bee.

Using elements of social capacity building and cohesive team building, two foremen were tasked with six apprentices and shown novel ways to up-cycle materials found on their worksite which would normally go into waste material. Workers and their families were actively engaged in transforming their site offices using innovative recycled office furniture and planter boxes (see Figure 3).

**Figure 4:**
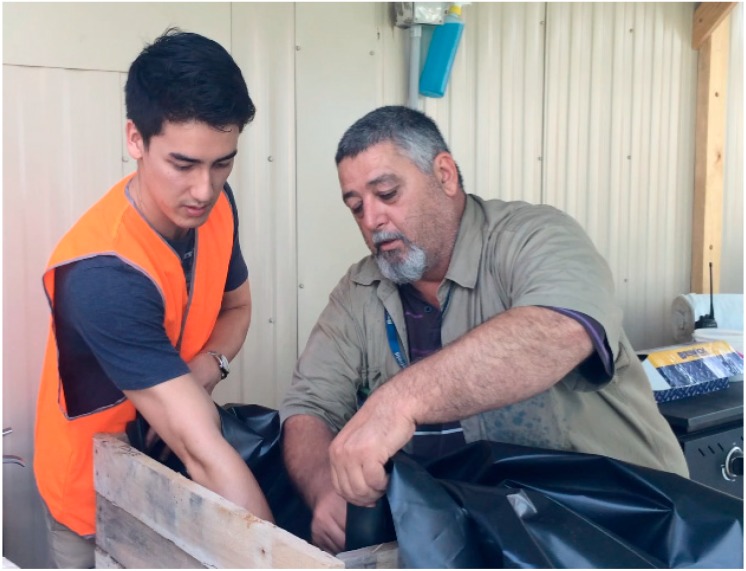
A foreman and apprentice working together during the working bee.

The use of the “foreman-apprenticeship” relationship reinforced team building and increased social engagement. One of the managers remarked on the day, “obviously there is a huge team building exercise going on”. Another site-manager commented: “they kind of jumped in and the community collaborative spirit… the tapping into a connectedness with the plants and some of the people and you could see a different side of it coming in”. In sum, the day was heralded as an exemplary team building exercise although this was a secondary objective (see Figure 4).

**Figure 5:**
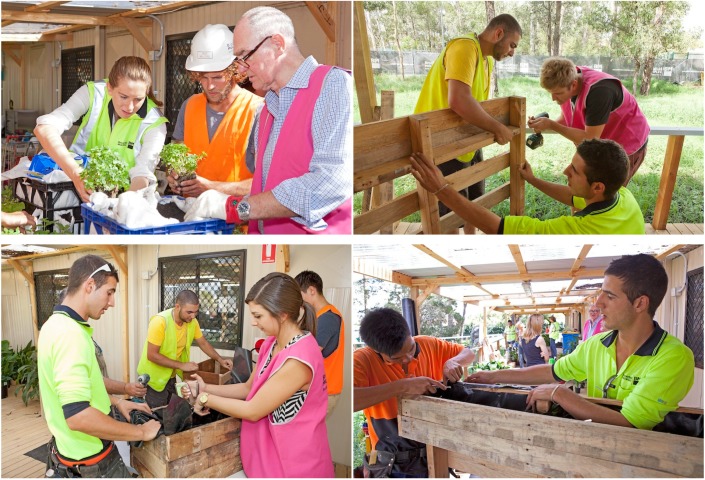

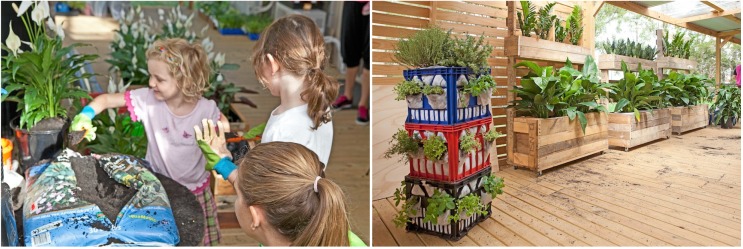
Collage of team building unfolding during working bee.

**Figure 6:**
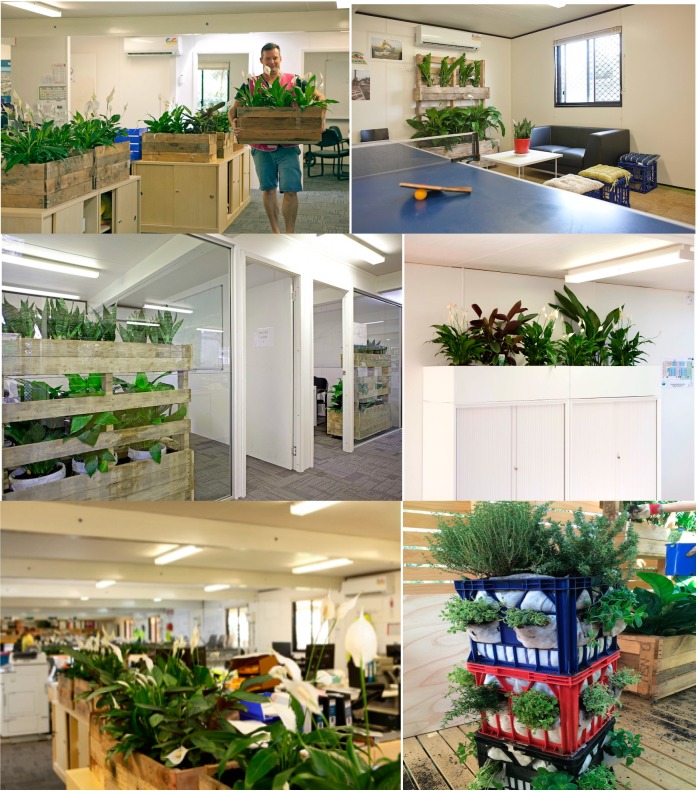
The plants enter the workplace.

Once the site workers were up-skilled by the foreman, they were responsible for the construction of their personalized recycled planter box, choice of greenery and general maintenance for their plants. Workers were interviewed whilst building vertical pallet trolleys and vegetable gardens from recycled materials. The artifacts produced are shown in the following collages (see Figure 5 and Figure 6).

### 4.3. Phase 3: In-Depth Site Office Interviews

Three weeks after the working bee day, the researchers conducted onsite individual interviews (15 min per subject) with randomly selected workers across all levels of management (n = 12) to ascertain the initial impact of the biophilic intervention upon their workspace. Researchers actively probed workers to encourage them to reflect deeply on their responses. All workers were receptive to this approach and forthcoming with measured and thoughtful dialogue.

Interview questions:

1. Demographics: (i) Gender and Age, (ii) Role or Position and (iii) Time spent in Site Office
2. Rate this site office against previous site offices you have worked in (score out of 10).
3. Does this office layout help or hinder the work environment at BM? Why/why not?
4. Do you think it is beneficial in terms of health and well-being? Why/why not?
5. Does this office do anything for you in terms of fatigue or stress? Why/why not?
6. What about creativity and mental acuity—any changes by being in this office? Why/why not?
7. In terms of collaboration and co-operation, have there been any noticeable differences? Why/why not?
8. Did you come to the working bee© If not, what was your initial impression when you walked into the office space the following Monday?
9. Name three qualities that best describe “the vibe” of this site.

The narratives were transcribed and emergent themes coded. Collated results are outlined in the following section.

## 5. Results and Discussion

Due to the nature of data collected during the interviews, observations and video footage, reviewing all of the explicate outcomes obtained in this phase is clearly beyond the scope of the paper. In addition, some emergent themes need to be confirmed during the ongoing longitudinal follow-up before they can be reported. However, our preliminary analysis of the results has revealed intriguing trends and insights. With regard to interview Question 1, a breakdown in the demographics of the 12 workers and their workplace information is provided in Table 3.

**Table 3:** A snapshot of the demographics of workers.

Gender	Age	Role/Position	Time Spent in Office
Female	25 years	Site Engineer	70%
Male	29 years	Services Manager	80%
Male	32 years	Project Manager	60%
Male	27 years	Site Engineer	40%–50%
Male	32 years	Senior Site Supervisor	20%–30%
Male	41 years	Site Manager	50%
Male	27 years	Foreman	20%
Male	22 years	Cadet	90%
Male	35 years	Contracts Manager	99%
Female	25 years	Site Secretary	100%
Male	31 years	Design Manager	95%
Male	31 years	Contract Administrator	90%

What stands out from this table is the relatively young age of people working in the site office, that the workers are predominantly male, and the variable amount of time that workers utilize this site office space. The site office space occupies the vast amount of working time for many of the staff on this building site.

Individuals were asked during interview Question 2 to rate (score out of 10: 1 = very poor, 10 = excellent) previous site offices they had worked in against the biophilic office. Table 4 illustrates their responses.

**Table 4:** Rate this office against previous offices you have worked in (score out of 10, 1 = very poor, 10 = excellent).

Respondent	Rating of Previous Office Site(s)	Rating of Biophilic Office
1	5	9
2	6–7	8–9
3	6	8–9
4	3	8
5	6	8–9
7	7–8	9
8	1–2	8
9	3–4	9
10	5	8

This data makes obvious that the transformed biophilic site office was considered by its workers to be superior to previous site(s), in some cases by a very wide margin. It must be acknowledged that a limitation of our study lies here in the lack of a pre-and post- intervention design. In asking workers to make a retrospective evaluation of previous site sheds as against the present modified site shed, results may indicate bias.

Data collected from interviews identified several emerging themes: the unique nature of this site office (compared to other site offices); sustainable workplaces and the transfer of learning from workplace to home (sustainable practices and ownership); high performance workplaces; impact of external surroundings of working site office; and the role and impact of “green space” (specific plants) in the workplace. For the purposes of this paper, two themes will be addressed: high performance workplaces and the role of plants in the workplace. The justification for the choice to focus on these elements is as follows: it was felt that since the initial interviews had been conducted so soon after the Working Bee (three weeks), the impact of that day may still have remained as a strong influencing factor on the workers’ responses. In addition, the researchers deduced that a longer time interval was required in order to tease out the nature of sustainable workplaces and if there was a transfer of learning from workplace to home. The role of the transformed green working space and the re-design in terms of open space collaborative spaces, seemed to suggest an immediacy of impact that should be reflected in the interviews.

### 5.1. High Performance Workplaces

The site office, although by its very nature transitory and temporary, is in fact the type of workplace setting that these workers and managers inhabit serially over their working lives in the construction industry. Although the site may be temporary, the workers are permanently in similar structures. Most workers noted an overwhelming positive difference between previous site offices and the present one:

“For me this is the best office that I’ve ever worked in, so an 8 or a 9 (rating out of 10)”.

“…and my last site, I didn’t want to go to the office. So out of 10, it would probably be 3”.

Our workers described previous site offices as “aggressive”, “stark surroundings”, “sterile environment” and “stale kind of environment”. As one participant elaborated:

“And if you look at most site offices, they’re fairly cold, harsh. Walled sort of environments, and that’s not the sort of environment that’s conducive to really collaborating well and just creating an atmosphere in which you can present your ideas strongly. But it doesn’t have to be an absolute sort of contest till the death to get those ideas across”.

In contrast, many viewed the biophilic site office as increasing social capability and improving workplace relations. Not only had the extra spaciousness of the working environment been noticed, but also the more “functional light and airy spaces” (Almusaed and Asaad [36]) had changed the dynamics. Several workers referred to the “softer feel” of the place, and one also identified the “softening interactions” that now took place in the site office. These included more communication between younger and older workers, between the more and less experienced members of the team:

“Sometimes I get stuck on certain things on site, and I come back to the office and you know, I’m looking for an answer first from someone. And I find that this office kind of gives me an opportunity to speak to different people. I’ll just walk past someone, and I’ll think to myself, maybe I can ask this person. You know, because you get an opportunity to see people in their open area. You’re not, you know,… sometimes if you’re in an office, you’re kind of restricted. Like people might not come up to you as much. But if you’re in an open area, you know, you kind of feel as though everyone’s on the same level in that sense, where you can walk past and just have a conversation and ask questions”.

According to workers, spontaneous collaborations occurred that previously would not have taken place in another site. For example, informal collaborations expedited problem solving across teams:

“…Between the site team and the design team. So sometimes, because they are based on site, and 85% of their time is spent on site, they don’t get that opportunity to maybe look at drawings and at that same time, collaborate with the design team who has spent, you know, a couple of weeks looking into certain issues such as the way things are built. And you know, to have that opportunity here in the office where they can grab someone, ask a question, … the problem can be solved in five min rather than let the problem be ongoing for a couple of days before they bring it to the attention of the design team”.

The newly designed open plan arrangement suited this type of increased social interaction, as well as opportunities afforded by the range of smaller meeting places:

“There was more room—that was definitely a bonus. I came from a previous site where our site shed was quite limited, and there wasn’t really a lot of room to kind of set up and have an area to work with. It was, yeah, very different. We also didn’t have a lot of meeting rooms, so a lot of the conversations where we didn’t want to make a lot of noise, we would walk outside and talk on the phone. Other areas would be, I think for me a kitchen, especially. It was just more a lunch area. We didn’t have that prior; it was just a small kitchen, and you had your lunch at your desk. So it gives a kind of opportunity for a bit of balance, without working from your desk, because normally you just go to your desk and just check your emails while you’re eating your lunch… So yeah, and because we’re in between site and office, sometimes when we do come back to the office, we kind of just need, I don’t know… Sometimes it’s nice to walk into the kitchen and sit down and just recollect your thoughts and think about what you need to do and make some calls. …Another thing was, I guess, just interaction with other people. There weren’t kind of shelves in front of you, so you could see the person next to you and talk to them. And yeah, I guess it was just a very different layout for a site office”.

Some workers found those increased interactions distracting, but these responses were in the minority:

“Personally, I haven’t got the best attention span anyway, so with everyone talking, especially, I’m in the design hub so there’s a constant flow of people coming in and asking questions. Sometimes, I find it very hard to concentrate, as in focus on reading the emails. A lot of what I do is reading reports and trying to knuckle down and get design done…I just literally was losing my temper a couple of seconds ago because I’m trying to read an email which is many pages of A4 long. I’ve printed it off. I can’t focus because there’s seven different conversations, and everyone asks you, because you’re sitting there, and it’s so open plan…”.

Still, the collaborative nature of the space was recognized by the same person:

“I think collaboration in this job has been good, and a lot of that’s been because of the way the office is set up. A lot of it’s been from the initiatives that certain people in the office have driven: with every Friday at 2 o’clock, we review something in the meeting room... Yeah, I think it has been good for collaboration”.

From natural lighting, furniture made with natural materials, white painted walls, and recycled carpet, to open windows and hearing bird sounds, all of the workers delineated different positive attributes of the unusual biophilic workspace. The workplace was enhanced from various perspectives. Early accounts from the qualitative interviews also suggest that this space increases social capacity and collaboration, and may lead to gains in productivity that we are exploring in the longer research project.

### 5.2. The Impact of Green Spaces in Workplaces

The dominant effect of the redesign of the site office seemed to be a change in the social dynamics of the space. However, the researchers were keen to tease out the specific impact of the plants, especially how workers perceived green elements in the design:

“I guess as soon as you walk into our office from the front gate, you notice something different because it’s got that vibe of you know, you’re kind of secluded away. And you know, you don’t really know what to expect, and then you walk in, and I think it looks a lot more, I don’t know, modern, relaxing. I’m not sure what words to use, but it’s different in a very positive way”.

This user does not speak about the “green” of the site office directly here, but the plants were mentioned by this subject subsequently:

“Yeah, when I walk to my desk, and you see plants, and you’re just, you know, it’s different. You don’t feel as though you’re indoors the whole day, if that makes sense”.

Another respondent who commented on his high stress levels in his last job, referred to the site office plants in this context:

But I do enjoy having them (the plants) now; I think it’s really nice. I’m not super-stressed at the moment. I’m in one of my calmer cycles, so I don’t know if that’s down to the plants, or whether that’s down to just where the job’s at. I like them.

This same worker identified his own need for natural surroundings in the workplace:

“I think natural light is really important; it’s one of my favourite aspects whenever we’re working on a job and designing a job. I think it’s important. Like I say, I really like the plants as well. I didn’t like the fertiliser smell for the first couple of days when they came in, but no, I like it, I do. I actually like the deck. I like there’s just a bit of a garden bit out the front; when you’re out there on the front, it’s nice. It’s nicer than what I’m used to. Last time I would sit outside the office on a roundabout pretty much making phone calls”.

Even the office worker who was least receptive of the biophilic intervention in the group acknowledged that the plants in some way contributed to the transformed working environment:

Look, I’m a bit of a sceptic to be honest with you, when it all sort of comes to this sort of stuff. But I was actually surprised when we did all the plants, because it actually, it is good. And it does make, it does create a better vibe within the office.

When questioned about the meaning of the word “vibe” this worker explained:

“I’d say “energetic” would be one word: relaxed, calm, enjoyable. You know, this is probably the best, yeah, probably the best office I’ve worked in”.

The extent to which the plants contribute to the ‘vibe’ is unclear in this respondent’s interview. From other interviews, the plants were clearly having an impact:

“What did I think? I thought, wow there’s plenty of plants in here—very different for a site office. I thought it looked good. I had no issues, no issues at all with it. I think it is good. You know, our subbies (subcontractors) come in and have a meeting with us, and they go, geez, where did you get all the plants? I don’t think it’s going to have a negative impact on anyone who works here, that’s for sure”.

And another states:

“Look, it may reduce stress and fatigue and stuff like that, but I think not knowing, it probably does. But it definitely takes away that sort of sterile environment that you sort of get in a lot of offices and that. So I may not directly know that it’s making me feel a lot better, but you walk in, and it doesn’t feel like your standard office”.

At this preliminary stage in the research, we note that green workplaces matter to workers who inhabit them in explicit ways, perhaps more so to those who inhabit temporary spaces such as site offices. Despite issues with responsibility for watering and maintaining the plants, we propose that the greenery itself contributes to a changed working environment:

“I suppose when you look up and see a bit of greenery around, it kind of reminds you you’re in a kind of living environment… We’re working long hours though; I’m sure it does help though, just not being such a stale kind of environment… Anything that’s natural, anything natural that’s I suppose growing and changing every day”.

Lastly, the researchers caution that generalisations to other populations at this point in time could be problematic due to the interviews being conducted within a few weeks of the “working bee” intervention. Clearly, issues such as “recency effects” and “honeymoon periods” could have favourably skewed the results. The future six-month interval data collection over the two years of the project will enable the researchers to gauge the enduring impacts and better understand the processes and mechanisms underpinning these claims. Notwithstanding these acknowledged limitations, the researchers posit that their preliminary results act as a benchmark for future comparisons.

## 6. Conclusions 

Historically, site workers have been housed in temporary, “match-box-sized” offices for the duration of construction projects, extending up to multiple years. These offices become the “norm” for their office space as workers transition from one construction site location to another. In this Brookfield Multiplex Australian study, aspects of open plan design and green interior spaces were purposefully infused into the newly devised bespoke office site.

Initial responses from occupants were clearly supportive of the newly introduced biophilic design elements to their workspace. Some benefits perceived by the workers using them included: enhanced collaboration amongst staff, including across teams, improved morale, and mitigation against stress. Even though this longitudinal study is ongoing for two years, early trends in responses from the first few weeks of occupancy suggested resounding approval by office workers. In short, the research is pointing towards a potential outcome that biophilic-designed site offices are linked to perceived social benefits and increased employee functioning including cooperation and mentoring, and to positive psychological effects, such as improved work satisfaction and higher morale. These are all characteristics of a high performance workplace and reinforce the findings of earlier research. What remains to be explored as the project continues are the effects of these design elements on the specific performance of workers and managers in their work spaces and the overall impact of plants in the workplace environment, including on non-subjective factors, such as employee retention and productivity. In addition, it remains to be seen to what extent the results are due to the biophilic elements or the impact of the Working Bee. Data collection currently ongoing until early 2016 will address a number of measures of workplace effectiveness and other quantifiable benefits, such as decreased absenteeism and improved mental acuity.

## References

[1] Burchett M., Torpy F., Brennan J., Craig A. (2010). Greening the Great Indoors for Human Health and Wellbeing.

[2] Dannenberg A., Frumkin H., Jackson R. (2011). Making Healthy Places: Designing and Building for Health, Well-Being, and Sustainability.

[3] Elings M., Hassink J., van Dijk M. (2006). People-plant interaction. Farming for Health.

[4] Frumkin H. (2001). Human health and the natural environment. Amer. J. Prevent. Med..

[5] Kaplan S. (1995). The restorative benefits of nature: Towards an integrative framework. J. Environ. Psychol..

[6] Kaplan R., Kaplan S., Francis M., Hester R. (1987). The garden as restorative experience: A research odyssey. Meanings of the Garden Conference Proceedings.

[7] Kaplan R., Kaplan S. (1989). The Experience of Nature: A Psychological Perspective.

[8] Kellert S. (2012). Birthright: People and Nature in the Modern World.

[9] Kuo F., Sullivan W. (2001). Environment and the Inner City: Does vegetation reduce crime?. Environ. Behav..

[10] Nielson T.S., Hansen K.B. (2007). Do green areas affect health? Results from a Danish survey on the use of green areas and health indicators. Health Place.

[11] Shoemaker C. (2002). Interaction by Design: Bringing People and Plants Together for Health and Well-Being.

[12] Wilson E.O. (2001). Nature matters. Amer. J. Prevent. Med..

[13] Berman M., Jonides J., Kaplan S. (2008). The cognitive benefits of interacting with nature. Psychol. Sci..

[14] Lewis C. (1973). People-plant interaction: A new horticultural perspective. Amer. Hortic..

[15] Lewis C. (1995). Human health and well-being: The psychological, physiological, and sociological effects of plants on people. Acta Hort..

[16] Lewis C. (1996). Lewis. Green Nature/Human Nature: The Meaning of Plants in Our Lives.

[17] Relf D. (1992). The Role of Horticulture in Human Well-Being and Social Development.

[18] Ulrich R., Kellert S.R., Wilson E.O. (1993). Biophobia, and natural landscapes. The Biophilia Hypothesis.

[19] Ulrich R. Influence of garden on health outcomes. Proceedings of American Society of Landscape Architects Annual Meeting, Therapeutic Gardens Forum, Missouri Botanical Garden.

[20] Ulrich R.S. Evidence-based garden design for improving health outcomes. Proceedings of Therapeutic Gardens Conference, University of Minnesota.

[21] Ulrich R., Parsons R., Relf D. (1992). Influences of passive experiences with plants on individual well-being and health. The Role of Horticulture in Human Well-Being and Social Development.

[22] Verderber S. (1986). Dimensions of Person-Window transactions in the hospital environment. Environ. Behav..

[23] Ottoson J., Grahn P. (2005). A comparison of leisure time spent in a garden with leisure time spent indoors: On measures of restoration in residents in geriatric care. Landscape Res..

[24] Mayer F.S., Frantz C.M., Bruehlman-Senecal E., Dolliver K. (2009). Why is nature beneficial? The role of connectedness to nature. Environ. Behav..

[25] Barton J., Pretty J. (2010). What is the best dose of nature and green exercise for improving mental health? A multi-study analysis. Environ. Sci. Technol..

[26] Lafortezza R., Carrus G., Sanesi G., Davies C. (2009). Benefits and well-being perceived by people visiting green spaces in periods of heat stress. Urban For. Urban Greening.

[27] Hassink J., van Dijk M. (2006). Farming for Health: Green-Care Farming across Europe and the United States of America.

[28] Haluza D., Schönbauer R., Cervinka R. (2014). Green perspectives for public health: A narrative review on the physiological effects of experiencing outdoor nature. Int. J. Environ. Res. Public Health.

[29] Wilson E.O. (1984). Biophilia.

[30] Wilson E.O. (1975). Sociobiology: The New Synthesis.

[31] Kellert S. (2005). Building for Life: Designing and Understanding the Human-Nature Connection.

[32] Kellert S. (2013). Occasional Address Graduation Speech.

[33] Kellert S.R., Wilson E.O. (1993). The Biophilia Hypothesis.

[34] Kellert S.R., Kahn P.H., Kellert S.R. (2002). Experiencing nature: Affective, cognitive, and evaluative development in children. Children and Nature: Psychological, Sociocultural, and Evolutionary Investigations.

[35] Meltzer N., Bobilya A., Mitten D. An investigation of the effect of an outdoor orientation program on participants’ biophilic expressions. Proceedings of 13th Annual Symposium on Experiential Education Research (SEER).

[36] Almusaed A., Almssad A. Biophilic architecture: The concept of healthy sustainable architecture. Proceedings of 23rd International Conference on Passive and Low Energy Architecture.

[37] Berry W. Nature Writing in America: The Place of Wendell Berry. http://numerocinqmagazine.com/2012/01/24/wendell-berry-nature-writing-in-america-by-adam-regn-arvidson/.

[38] Lohr V., Pearson-Mims C., Goodwin G. (1996). Interior plants may improve worker productivity and reduce stress in a windowless environment. Environ. Hort..

[39] Bringslimark T., Hartig T., Patil G.G. (2007). Psychological benefits of indoor plants in workplaces: Putting experimental results into context. HortScience.

[40] Dijkstra K., Pieterse M.E., Pruyn A. (2008). Stress-reducing effects of indoor plants in the built healthcare environment: The mediating role of perceived attractiveness. Prev. Med..

[41] Grinde B., Grindal Patil G. (2009). Biophilia: Does visual contact with nature impact on health and well-being?. Int. J. Environ. Res. Public Health.

[42] (2000). Healthy Buildings, Healthy People: A Vision For the 21st Century.

[43] (2003). Indoor Air Quality and Student Performance, Report of Indoor Environments Division.

[44] 202020 Vision Brookfield Multiplex. http://202020vision.com.au/partner/?id=1437.

[45] Mayer S., Frantz C. (2004). The connectedness to nature scale: A measure of individuals’ feeling in community with nature. J. Environ. Psychol..

